# A child infected with severe acute respiratory syndrome coronavirus 2 presenting with diarrhea without fever and cough: A case report

**DOI:** 10.1097/MD.0000000000021427

**Published:** 2020-08-14

**Authors:** Qian Liu, Yong Zhang, Yuan Long

**Affiliations:** Departments of cardiovascular, Wuhan Children's Hospital(Wuhan Maternal and Child Healthcare Hospital), Tongji Medical College, Huazhong University of Science and Technology, Hubei, China.

**Keywords:** diarrhea, nucleic acid amplification test, pneumonia, severe acute respiratory syndrome coronavirus 2

## Abstract

**Rationale:**

Coronavirus disease 2019 (COVID-19), now a global pandemic, has spread to a large number of countries around the world. Symptoms of COVID-19 can range from mild to severe, including fever, cough, shortness of breath, and pneumonia. Some cases even remain asymptomatic. Data regarding the epidemiological and clinical features of children with COVID-19 are limited. Symptoms in children are thought to be atypical when compared with adults. As a result, diagnosis in many children is likely to be missed. Children presenting with atypical symptoms, especially those with a history of exposure, should be referred to early screening.

**Patient concerns:**

A 23-month-old boy presented with a 2-day history of diarrhea. Chest computed tomography scan showed pneumonia. After admission to the hospital, the patient exhibited no diarrhea or other symptoms. Positive presence of severe acute respiratory syndrome coronavirus 2 (SARS-CoV-2), the virus that causes COVID-19, was confirmed by 5 consecutive rounds of nucleic acid amplification testing of nasopharyngeal swabs. The patient was also found to have liver damage.

**Diagnosis:**

Swabs were obtained for detection of SARS-CoV-2 RNA by established methods.

**Interventions:**

Chinese traditional medicine prescription

**Outcomes:**

Following treatment, signs of pneumonia on computed tomography scans were observed to be partially absorbed, and 2 consecutive rounds of nucleic acid amplification testing of swab samples were negative. The patient was discharged on the 21st day after admission to the hospital. On the 21st day after discharge, the patient had no recurrence of disease, no recurrence of pulmonary lesions, and normal liver function.

**Conclusion:**

This case study suggests that diarrhea not explained by common causes, such as acute gastroenteritis, could be a preliminary symptom of SARS-CoV-2 infection in children. Despite the lack of the presence of a fever or cough, lung pulmonary lesions were present in this child. SARS-CoV-2 infection may also cause hepatic injury. Even during the SARS-CoV-2 pneumonia recovery period, IgM and IgG antibodies can be positive for a long time.

## Abstract 2

Supplemental Digital Content is available in the text

## 1 Introduction

Coronavirus disease 2019 (COVID-19) is caused by severe acute respiratory syndrome coronavirus 2 (SARS-CoV-2). SARS-CoV-2 is genetically similar to the coronavirus that was responsible for the SARS outbreak in 2003.^[1]^ SARS-CoV-2 originated in bats, which are a natural reservoir for the virus. The virus then mutated and infected pangolins, which became an intermediate host of the virus.^[2]^ In late 2019, SARS-CoV-2 mutated again before being transmitted to humans.^[3]^ Symptoms of COVID-19 can range from mild to severe, including fever, cough, shortness of breath, and pneumonia.^[3–5]^ However, some patients infected with SARS-CoV-2 can remain asymptomatic, and symptoms such as diarrhea that are not currently part of the clinical diagnosis may be underestimated.^[6]^ Furthermore, data regarding the epidemiological and clinical features of COVID-19 in children are limited.

Wuhan Children's Hospital, the only center assigned by the central government to treat SARS-CoV-2 infected children under the age of 16 years in Wuhan, has confirmed more than 500 cases. Here we report the case of a child infected with SARS-CoV-2 presenting with diarrhea without cough and fever.

## 2 Case report

**Figure 1:**
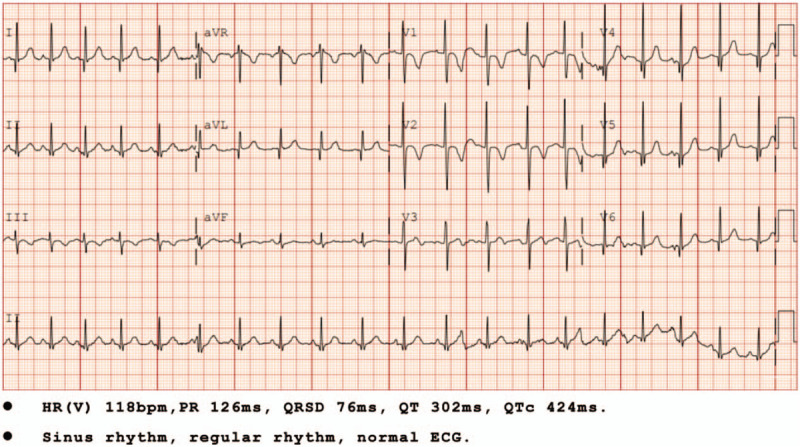
Electrocardiogram 1st day of admission. HR(V) 118bpm,PR 126ms, QRSD 76ms, QT 302ms, QTc 424ms. Sinus rhythm, regular rhythm, normal electrocardiogram.

**Figure 2:**
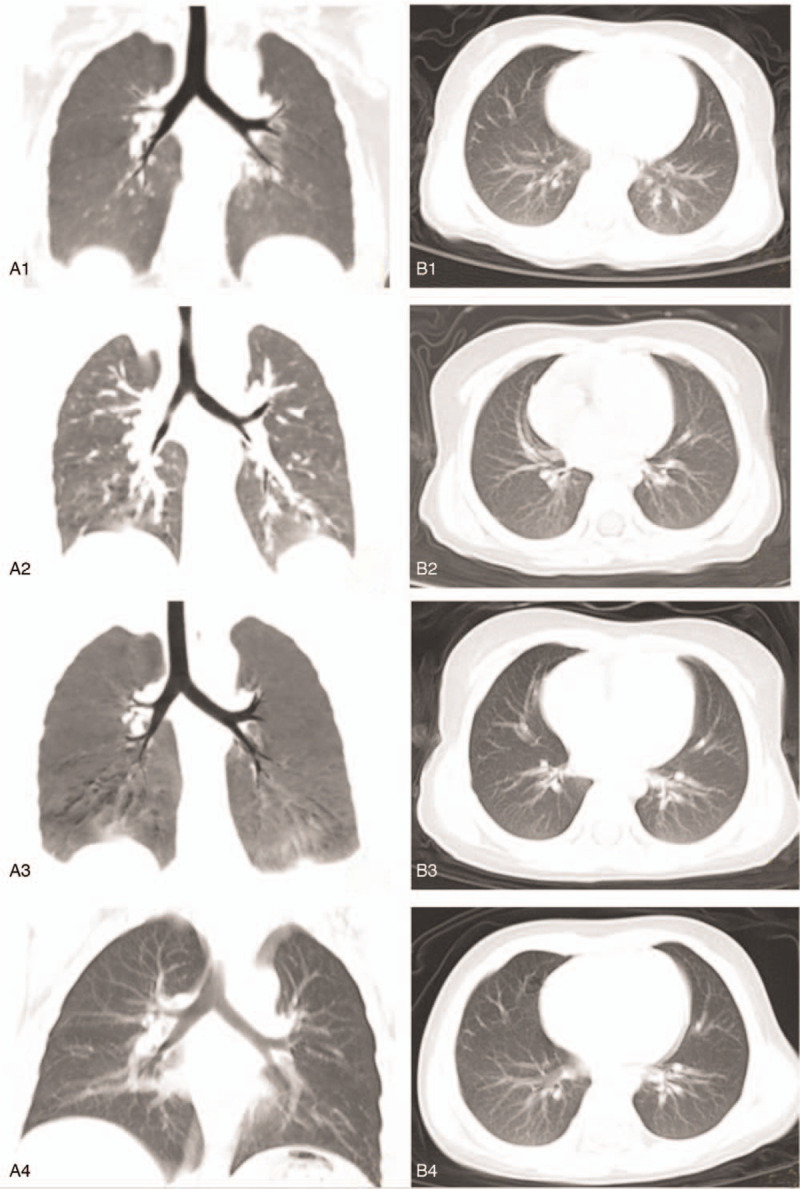
Chest CT. 1st day of admission (A_1_, B_1_). A_1_ Mediastinal window: Thymus shadow size and density are normal. The trachea and bronchi are unblocked. No displacement was observed in the mediastinum. B_1_ Lung window: Increased of lung markings. Small flaky fuzzy shadows can be seen in the right lower lobe of the lung. 19th day of admission (A_3_, B_3_). A_2_ Mediastinal window: As before. B_2_ Lung window: Fibrous stripes can be found in the upper lobe of right lung. The lesion in the lower lobe of the right lung has been absorbed. 4th day after discharge (A_3_, B_3_). A_3_ Mediastinal window: As before. B_3_ Lung window: The Fibrous stripes in the upper lobe of right lung just as before. 21th day after discharge (A_4_, B_4_). A_4_ Mediastinal window: As before. B_4_ Lung window: The Fibrous stripes in the upper lobe of right lung also as before. CT = computed tomography.

A 23-month-old boy presented with a 2-day history of diarrhea without cough, fever, shortness of breath, nausea, or vomiting. Physical examination was normal. The child's mother reported that the child had a typical birth, no past surgeries, and a negative family history of genetic disease. At the time of admission, the child's mother appeared healthy. However, she was diagnosed with COVID-19 7 days after her child was admitted to the hospital. Her primary symptoms were a fever and cough lasting for nine days. Subsequent to hospital admission, the child exhibited no diarrhea or other clinical symptoms. Laboratory tests performed over 21 days following admission of the child included the following: routine blood examination (Table 1), infection markers (Table 1), biochemical indicators (Table 1), SARS-CoV-2 test (Table 2), etiological examination (Table 3), immune function and Treg cells count (Table 3), immunoglobulin (Table 3), levels of cytokines (Table 3), EGG (Fig. 1), and chest computed tomography (CT) (Fig. 2 A_1,_ B_1)_. Chest CT showed pneumonia. However, the most common abnormal CT finding of ground glass opacities in adults with COVID-19 was not observed (Fig. 2 A_1,_ B_1)_. On the 2nd day of hospital admission, a positive SARS-CoV-2 infection was confirmed by nucleic acid amplification testing (NAAT) of nasopharyngeal swabs (Table 2). On the 6th day of admission, biochemical indicators suggested impaired liver function (Table 1). The patient was treated with a Chinese traditional medicine prescription (Table 4) suitable for both the treatment of pneumonia and liver damage. After days of treatment, laboratory indicators were reviewed. On the 13th day after admission, laboratory evaluation showed normal liver function (Table 3). On the 19th day after admission reviewed Chest CT showed no new pulmonary lesions (Fig. 2 A_2,_ B_2_), and blood tests showed normal (Tables 1 and 3). But subsequent NAAT, repeated 4 times, indicated positive SARS-CoV-2 infection in the child (Table 2). Finally, the patient was discharged on the 21st day after admission to the hospital. Before discharge chest CT showed no recurrence or new pulmonary lesions other than pulmonary fibrosis in the upper lobe of the right lung (Fig. 2 A_2,_ B_2_), and NAAT, repeated two times, was also negative (Table 2). On the 4th day after discharge, the patient had no new pulmonary lesions (Fig. 2 A_3,_ B_3_) and normal liver function (Table 3). A new method that was not available during the time of hospitalization was utilized to test SARS-CoV-2 by antibody detection on the 21st day after discharge (Table 5). Chest CT (Fig. 2 A_4,_ B_4_) and blood tests (Table 1) were reviewed at the same time. All results showed normal liver function with no recurrence or new pulmonary lesions. However, the antibody detection was positive for both IgG and IgM.

**Table 1:**
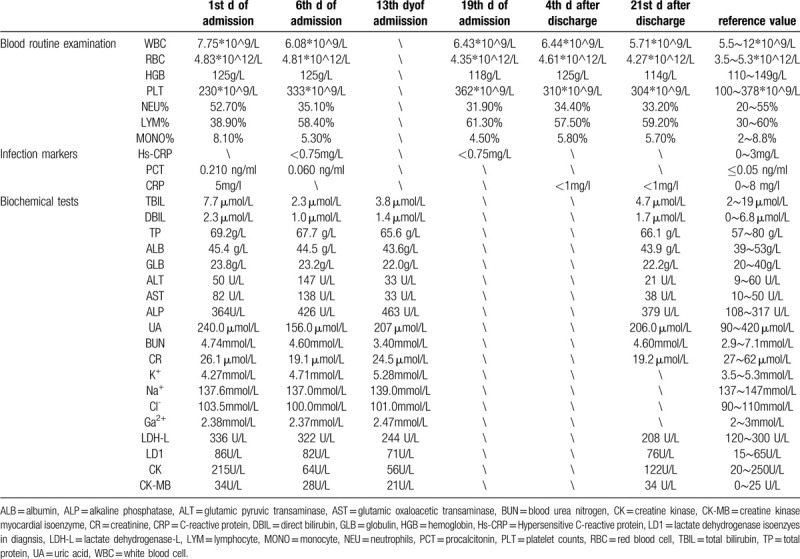
Blood tests 1.

**Table 2:**
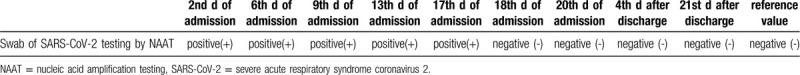
Swab of SARS-CoV-2 testing.

**Table 3:**
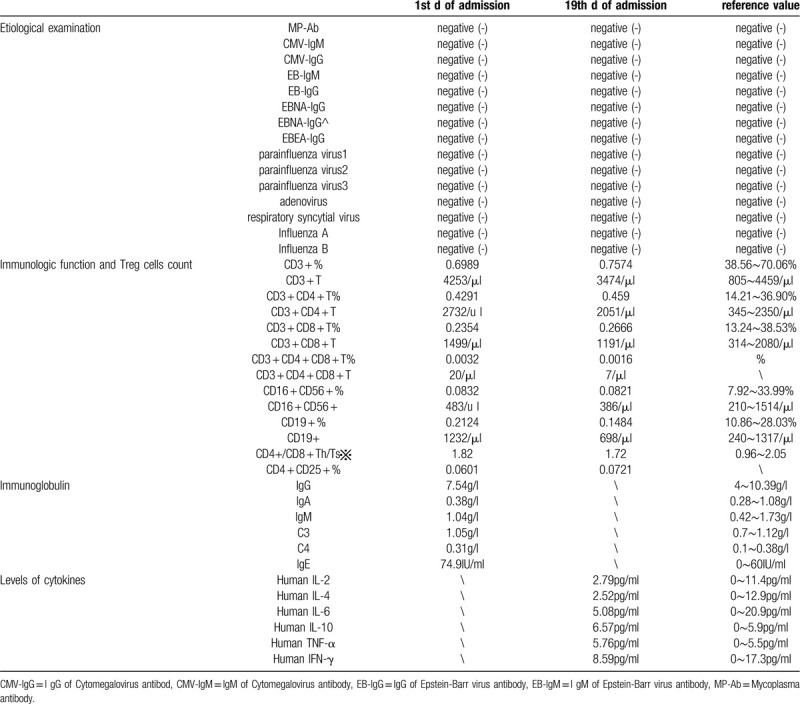
Blood tests 2.

**Table 4:**
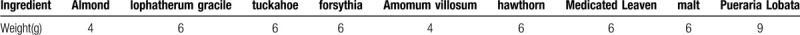
Chinese traditional medicine prescription.

**Table 5:**
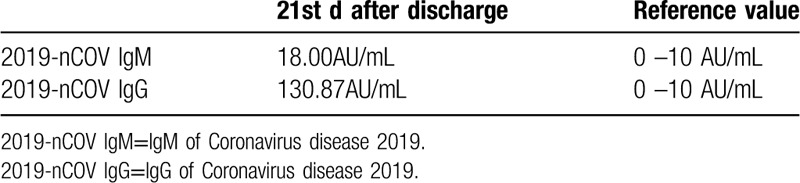
Antibody detection in blood.

The father of the patient signed an informed consent form, and the study was approved and monitored by the ethics committee of Wuhan Children's Hospital.

## 3 Discussion

Diarrhea is 1 of the most common symptoms of COVID-19 in children. COVID-19 can range from a temporary, mild condition to a potentially life-threatening 1. The case study described here presents a child living in Wuhan at the heart of the COVID-19 endemic, who was exposed to a mother with the classic symptoms of cough and fever. While his only clinical symptom upon arrival to the hospital was diarrhea, subsequent testing confirmed SARS-CoV-2 infection with a positive CT scan for pneumonia. Research has suggested a mutual interaction between SARS-CoV-2 and angiotensin-converting enzyme 2 (ACE2). SARS-CoV-2 is thought to disrupt the function of ACE2 and result in diarrhea.^[6]^ These studies are based on the evidence that SARS-CoV-2 and ACE2 share the same spike protein.^[7,8]^ In terms of the pathological importance of ACE2 in modulating intestinal inflammation and diarrhea.^[9]^This case study shows that children with SARS-CoV-2 infection can present with neither a fever nor cough despite the presence of lung pulmonary lesions. Diarrhea, lasting for just 2 days, can be the only symptom.

Furthermore, from this case it can be seen that routine blood tests and chest CT results are atypical in children with COVID-19 when compared with adults. In adults with COVID-19, blood tests often show increased lymphocytes with a decreased or normal white blood cell count.^[4]^ The 3 primary chest CT findings of SARS-CoV-2 infection in adults are ground-glass opacities, consolidation, and crazy paving. These can be seen in isolation or in combination with 1 another.^[10]^ Ground glass opacities are usually the first and the most common abnormal CT finding in adults with COVID-19.^[10]^ Portions of the lungs appear hazy gray, instead of black, with white markings for blood vessels. If the patient has a severe or more advanced SARS-CoV-2 infection, more fluid will accumulate in the lobes of the lungs resulting in a solid white appearance on a CT scan as the ground glass opacities consolidate. Finally, what is known as a crazy paving pattern can be seen on a CT scan. This pattern results from the swelling of the interstitial space along the walls of the lung lobules, which makes the walls appear thicker. It has been found that disease severity is proportionate to CT findings; patients exhibiting the most symptoms also exhibit the most severe findings on chest CT.^[10]^ In contrast to adults, most children with COVID-19 appear to have a milder clinical course.^[11]^ In the case study described above, pneumonia was confirmed by chest CT in the early stages of the disease (Fig. 2 A_1,_ B_1)._ This pneumonia was not severe and similar to pneumonia observed in the absence of COVID-19. With the development of the disease in adults, pulmonary fibrosis normally occurs (Fig. 2 A_2,_ B_2,_ A_3_, B_3,_ A_4_, B_4_). Prior to this case study, to our knowledge there have been no reports of the CT findings of SARS-CoV-2 in children. While preliminary, this case study suggests that the observed lung changes in children with COVID-19 may be mild and atypical in comparison to those observed in adults.

Upon examination, we confirmed that the child observed in this case study exhibited liver damage. We speculate that SARS-CoV-2 infection may cause hepatic injury. However, the mechanisms leading to this damage remain unknown. We also speculate that prompt treatment can effectively promote the recovery of liver function. In China, a traditional Chinese medicine prescription (Table 4) is widely used in the treatment of SARS-CoV-2-related pneumonia and its complications. The composition of the traditional Chinese medicine prescription is jointly researched and produced by the expert group of traditional Chinese medicine. The prescription chosen for the child in this case study was aimed at repairing pulmonary lesions and liver injury.

On the 21st day after discharge from the hospital, the patient had no recurrence of disease or new pulmonary lesions. However, antibody testing was positive for both IgG and IgM. Antibody detection is recently a new application for SARS-CoV-2 testing in Wuhan Children's Hospital. Researchers have found that serological IgM and IgG antibodies are detected as early as the 4th day after disease onset. However, the seropositive rate of IgM can increase gradually with IgG increasing sharply on the 12th day after onset.^[12]^ The researchers also found that IgM and IgG antibodies against SARS-CoV-2 could be detected in the middle and later stages of the disease. Furthermore, ELISA-based IgM and IgG antibody tests for serodiagnosis of SARS-CoV-2 have great specificity.^[12]^ In this case study, antibody detection was undertaken on the 44th day after onset of the disease. The patient was isolated both in and out of the hospital. Therefore, the chance of reinfection was extremely low. Given the current COVID-19 pandemic, IgM/IgG antibody test was recommended by WHO to provide an effective complement to nucleic acid tests for SARS-CoV-2 infection to confirm COVID-19 diagnosis.^[13]^

## 4 Conclusion

Compared with adults, children with COVID-19 may have mild or atypical symptoms resulting in a missed diagnosis and delayed therapy.^[11]^ Children with atypical symptoms, especially those with a history of exposure, should be referred to early screening. SARS-CoV-2 infection may cause liver damage, and timely intervention is beneficial to liver function recovery. Absence of clinical symptoms does not mean the absence of organ injury. Antibody detection is helpful even during the SARS-CoV-2 pneumonia recovery period. IgM antibodies can be positive for a long time. IgM/IgG antibody tests could offer a promising method to judge the infectiousness of patients with SARS-CoV-2 and give us a better understanding of COVID-19.

## Author contributions

All of the authors have accepted responsibility for the entire content of this submitted manuscript and approved submission.

Writing–original draft: Qian Liu

Writing–review and editing: Yong Zhang, Yuan Long.

## Supplementary Material

Supplemental Digital Content
