# Supplementary material for: A child infected with severe acute respiratory syndrome coronavirus 2 presenting with diarrhea without fever and cough: A case report
Source: Medicine (Baltimore). 2020 Aug 14;99(33):e21427. doi: 10.1097/MD.0000000000021427 (PMC7437845; doi:10.1097/MD.0000000000021427)

**Patient consent form**

**For a patient’s consent to publication of information about them in paper.**

Subject matter of photograph or article: A SARS-CoV-2 infected child presented with diarrhea

Journal name: MEDCINE

Manuscript number: MD-D-20-02756

Title of article: A SARS-CoV-2 infected child presented with diarrhea without fever and cough

Corresponding author: Qian Liu，M.D.


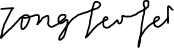


I [insert full name] give my consent for this information about MYSELF/MY CHILD OR WARD/MY RELATIVE [circle correct description] relating to the subject matter above (“the Information”) to appear in the journal and associated publications*.

I understand the following:

(1) The Information will be published without my name attached and The paper will make every attempt to ensure my anonymity. I understand, however, that complete anonymity cannot be guaranteed. It is possible that somebody somewhere - perhaps, for example, somebody who looked after me if I was in hospital or a relative - may identify me.

(2) The text of the article will be edited for style, grammar, consistency, and length.

(3) The Information may be published in the journal, which is distributed worldwide. The journal goes mainly to doctors but is seen by many non-doctors, including journalists.

(4) *The Information may also be used in full or in part in other publications and products published. This includes publication in English and in translation, in print, in electronic formats, and in any other formats . In particular the Information may appear in local editions of the journal or other journals and publications published overseas.

(6) I can revoke my consent at any time before publication, but once the Information has been committed to publication (“gone to press”) it will not be possible to revoke the consent.


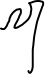

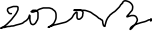

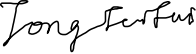


Signed:__________________________________ Date: _______________________


Supplement: Supplemental Digital Content [file medi-99-e21427-s001.docx]
